# Insights into periodontal disease: comparative analysis of animal models

**DOI:** 10.3389/fdmed.2025.1560101

**Published:** 2025-04-25

**Authors:** Binapani Barik, Saurabh Chawla, Bhabani Sankar Satapathy, Swadesh Kumar Pattanik, J. Aravind Kumar, Saleh Al-Farraj, Gurudutta Pattnaik, Mika Sillanpää

**Affiliations:** 1 School of Pharmacy and Life Sciences, Centurion University of Technology and Management, Khordha, Odisha, India; 2 School of Biological Sciences, National Institute of Science Education and Research (NISER), Khordha, Odisha, India; 3 Homi Bhabha National Institute (HBNI), Training School Complex, Anushaktinagar, Mumbai, India; 4 GITAM School of Pharmacy, GITAM Deemed to be University, Hyderabad Campus, Telangana, India; 5 Chitkara College of Pharmacy, Chitkara University, Punjab, India; 6 Saveetha School of Engineering, Saveetha Institute of Medical and Technical Sciences, Saveetha University, Chennai, Tamil Nadu, India; 7 College of Science, King Saud University, Riyadh, Saudi Arabia; 8 Saveetha School of Engineering, Saveetha Institute of Medical and Technical Sciences, Saveetha University, Chennai, Tamil Nadu, India; 9 Centre of Research Impact and Outcome, Chitkara University Institute of Engineering and Technology, Chitkara University, Rajpura, Punjab, India

**Keywords:** periodontal disease, animal model, bacterial infection, comparative study, limitations

## Abstract

Periodontal disease is a progressive condition characterized by the degradation of gingival tissues, periodontal ligaments, and alveolar bone, often resulting in tooth loss if untreated. Its pathogenesis is influenced by bacterial infections, host immune responses, and environmental factors. While human cell cultures provide insights into cellular mechanisms, animal models play a crucial role in understanding the complex host-pathogen interactions and developing therapeutic interventions. Various species, including rodents, dogs, non-human primates, and mini-pigs, have been employed in periodontal research due to their anatomical and immunological similarities to humans. These models allow the study of disease progression, systemic effects, and potential treatments in a controlled environment. However, challenges such as anatomical differences, ethical concerns, and the difficulty of accurately replicating human periodontal disease remain. Despite these limitations, animal models are indispensable for advancing periodontal research, offering insights into disease mechanisms and contributing to the development of novel therapies. This review evaluates the strengths and limitations of several animal models used in periodontal disease studies, emphasizing the need for further refinement to enhance their relevance to human conditions.

## Highlights

- Comprehensive Review of Animal Models
- Relevance to Human Pathophysiology
- Emerging Models and Technologies
- Model-Specific Strengths and Limitations
- Clinical Translation

## 1 Introduction

**Figure 1:**
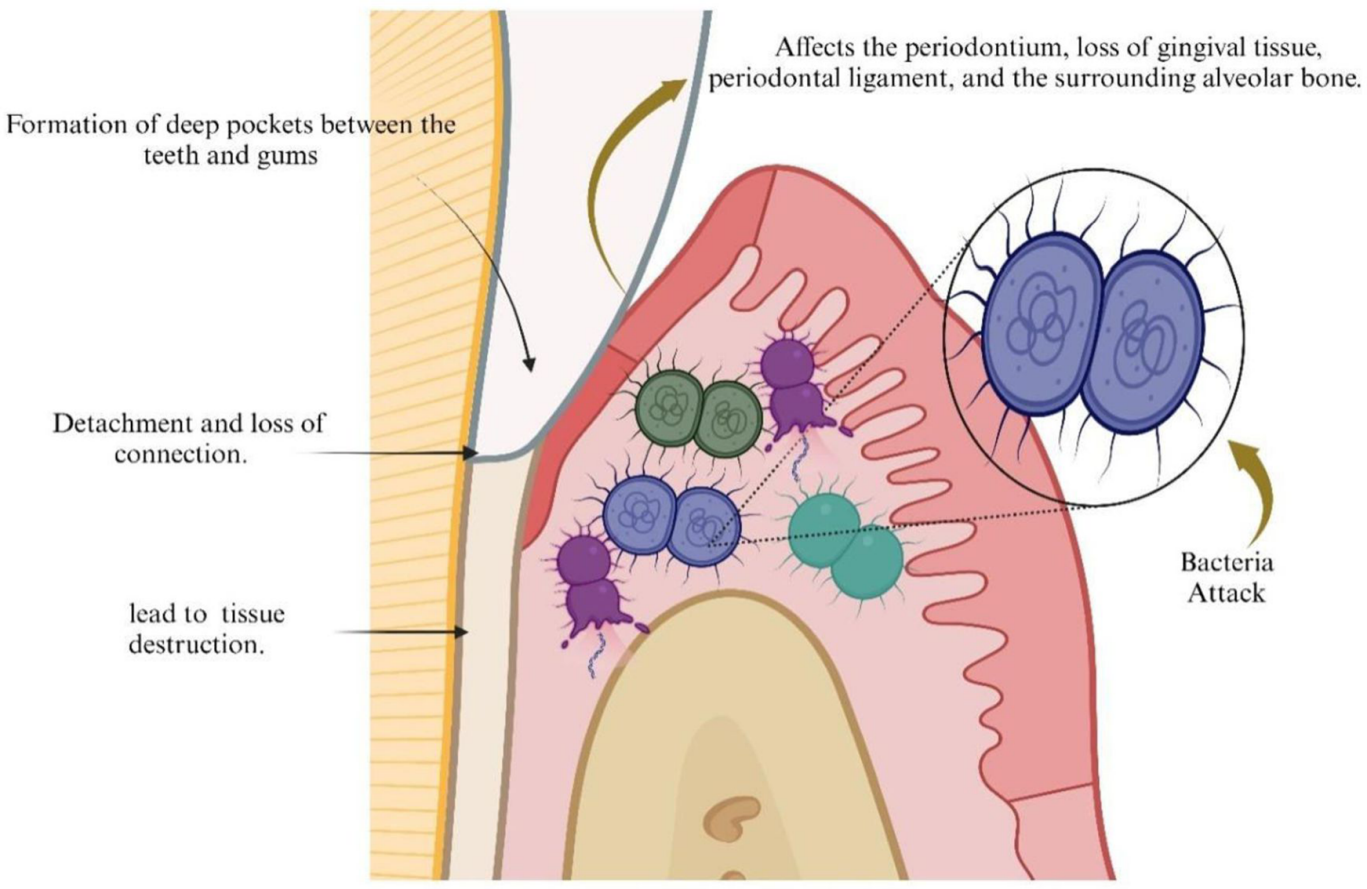
Periodontal conditions, including the formation of deep pockets and tissue damage. Created by Biorender.

Periodontitis is a common and long-term disease that affects the periodontium, causing the gradual loss of gingival tissue, the periodontal ligament, and the surrounding alveolar bone (1). The progressive and gradual decline of periodontal tissues may causes detachment, resulting in the formation of deep pockets between teeth and gums, potentially leading to infection and subsequent tissue degradation (2). The dental biofilm is formed often consists of a fraction of the gram (-ve) anaerobic commensally microbiota, together with opportunistic pathogens found in the oral cavity, such as *Porphyromonas gingivalis* (*P. gingivalis*) (3). There is an increase of polymorphonuclear cells (PMNs) at the site after infection and inflammation. In response to periodontal infections, PMNs emit reactive oxygen species (ROS), particularly superoxide, over the course of the illness (4). This oxidative burst aids in the destruction of bacteria and helps to resolve the infection. However, excessive ROS production can also lead to tissue damage and contribute to the progression of periodontal disease. In addition to causing tissue damage, excessive ROS production can also trigger an inflammatory response that further exacerbates periodontal disease. This chronic inflammation can eventually lead to the destruction of the supporting structures of the teeth, resulting in tooth loss if left untreated. Therefore, it is important for the body to strike a balance in ROS production to effectively combat infections without causing harm to surrounding tissues. The periodontal conditions, which include tissue damage and the development of deep pockets, are depicted in Figure 1. Moreover, PMNs release proteinases and other degrading enzymes that might damage the host tissues. In general, the oxidising chemicals lead to oxidative damage to the tissue in the gums, adjoining ligaments in the mouth, and also trigger the breakdown of bone by osteoclasts (5).

**Figure 2:**
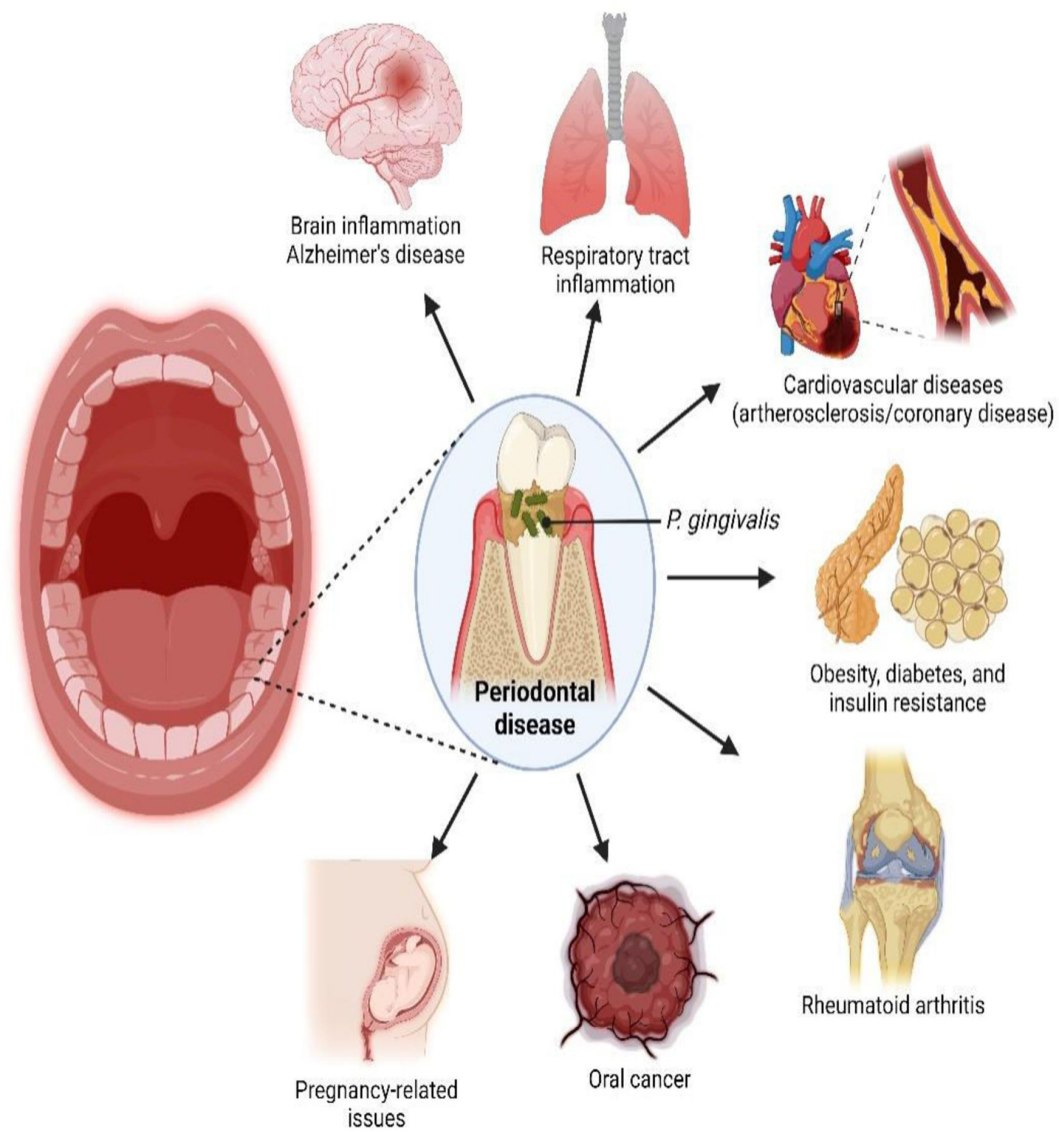
Systemic disorders that are linked to periodontal disease. Reproduced with permission from “Link between P. gingivalis, an oral pathogen, with various systemic diseases” by Yoke Chan Chow et al., licensed under CC BY 4.0.

Furthermore, periodontitis has been associated to systemic disorders such as cardiovascular disease, rheumatoid arthritis, and pregnancy related complications. The associated systemic diseases of periodontal disease are depicted in Figure 2. The development of chronic inflammation in the periodontium is triggered by intricate subgingival biofilms that may contain several potential periodontal infectious pathogens (5). The substances that are released during the progression of disease may trigger inflammation that may lead to release of multiple cytokines including interleukin (IL)-1ß, IL-6, and tumour necrosis factor (TNFα). These and other related biomolecules are consistently higher in the gingival crevicular fluid (GCF) and tissues of individuals with periodontitis. Levels of these inflammatory chemicals usually decrease after periodontal treatment (6).

The progression of periodontitis is not just dependant on bacterial infection, but it can also be linked to variations in an individual's sensitivity along with other factors such as race/ethnicity, poverty level and education (7). The population diversity in human beings and variation in individuals' response to infection plays a crucial role in the development and progression of periodontal diseases (8). To study the mechanism, progression and disease pathology of periodontal disease a suitable model system needs to be developed. Human cell cultures have shown to be valuable tools for studying certain specific aspects of the periodontal disease at the cellular level, they do not offer extensive information about the intricate host response.

To address these shortcomings animal models are extensively used in the area of biomedical research and study of periodontal disease. Therefore, animal studies offer a powerful tool for the examination of the host response which is crucial for developing improved therapies.

## 2 Models based on animals

**Figure 3:**
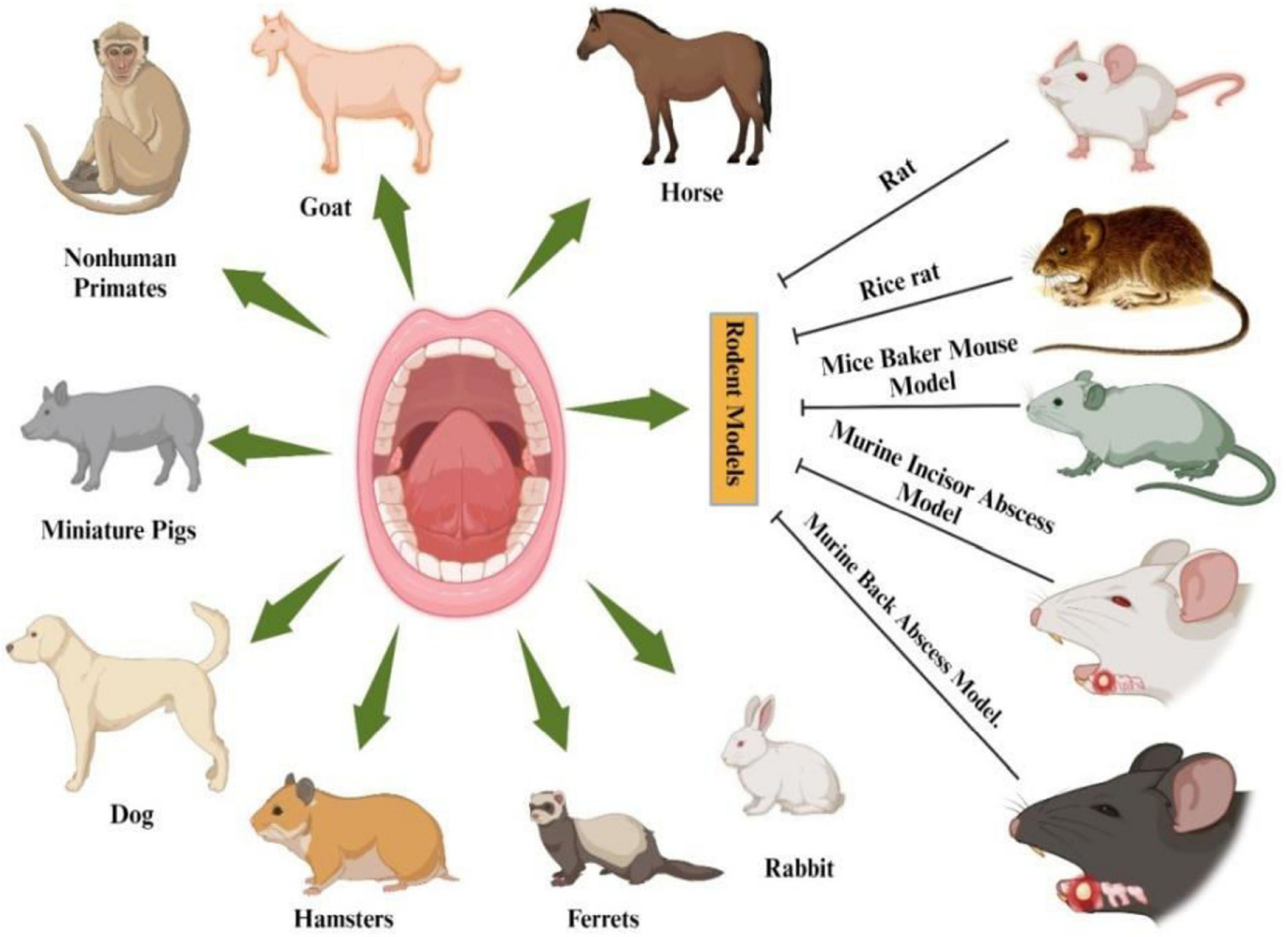
Established animal models for scientific examination of periodontal disease. Created by Biorender.

Animal models have endured a significant role in advancing our understanding of biological sciences, and similarly in the field of periodontology. Different species have been utilized to investigate the development of periodontitis and evaluate different treatment methodologies (9). Periodontal disease is one of the most frequent oral diseases in few animal species, (10). Periodontal disease may occur spontaneously or be deliberately produced in animals for experimental purposes. Like humans the primary clinical signs of periodontal disease in pet animals are bad breath, plaque and calculus accumulation; red, swollen, and bleeding gums, gingival recession; periodontal pocketing, bone loss, furcation exposure, mobile teeth leading to eventual loss of teeth making them suitable for modelling the human disease (11). The animal models of periodontal disease that are currently available for scientific investigation are shown in Figure 3.

Although animal models have yielded a substantial volume of data, it can be challenging to ascertain their relevance to humans. A straightforward and precise model that accurately replicates the development of periodontal disease in humans has not yet been identified.

### 2.1 Experimentally induced animal models of periodontal disease

There are certain animal models where the periodontal disease is experimentally induced to study the progression of disease, its pathophysiology and treatment options.

#### 2.1.1 Rodent models

In order to complement studies conducted on primate and human periodontal tissues, rodents offer a number of distinctive characteristics that can be used to evaluate microbial and host responses. These models are cost-effective, easy to handle, and have a short reproductive cycle, allowing for efficient data collection and analysis (12). Additionally, rodents can be genetically manipulated to mimic specific diseases or conditions, providing valuable insights into the mechanisms underlying periodontal disease. In each quadrant, rodents have three molars and just one incisor. By placing ligatures in the gingival sulcus around the molar teeth and increasing biofilm buildup and disturbing the gingival epithelium, studies on rats have been able to induce disease and accelerate osteo-clasto-genesis and bone loss. To establish the virulence of these species in rodents, other models involve the oral infection of these animals with specific human infections (13). With these methods, it is also possible to use strains that have had their genes changed in order to find specific parts of the host response and explain how those parts cause the illness. In recent times, several researchers have employed chemicals, microbes, or their byproducts to induce periodontal disease in gingival tissue. These approaches have provided valuable insights into the mechanisms underlying periodontal disease and potential therapeutic targets (14). Overall, these models have significantly advanced our understanding of the pathogenesis of periodontal disease and potential treatment strategies. Furthermore, these experimental models have also allowed researchers to test the efficacy of various treatment options, such as antibiotics, anti-inflammatory agents, and probiotics, in managing periodontal disease.

#### Rats

Rats are frequently used in experimental models of periodontitis due to similarities in periodontal architecture with humans, particularly in the molar region. Among rat models, the rice rat (*Oryzomys palustris*) is especially valuable due to its high susceptibility to naturally occurring and diet-induced periodontal disease, making it a suitable model for studying disease progression. Additionally, the species supplements its diet with animal matter such as insects, small crustaceans, mollusks, and occasionally fish or amphibians (15). Periodontitis was generated in rats by inserting a bacterial plaque retentive silk or cotton ligature into the gingival sulcus around the molar teeth. Furthermore, the injection of *p. gingivalis* has caused alveolar bone loss. Overall, rat models provide valuable insights into the mechanisms of periodontal disease progression and treatment strategies (16).

**Figure 4:**
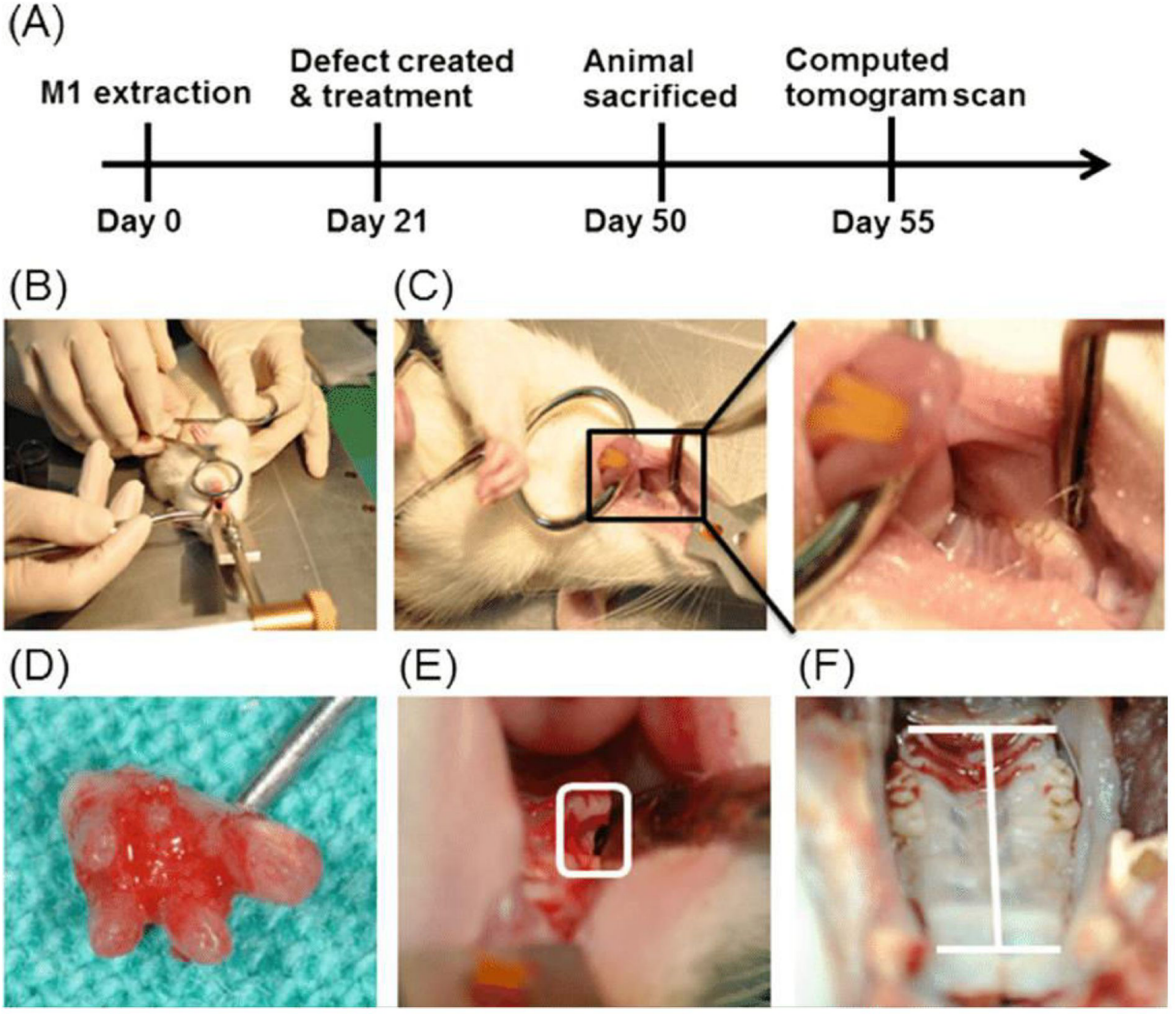
Displays a rat animal model used for studying periodontal diseases and the formation of osseous abnormalities. **(A)** Overall research methodology. The animals were categorized into control groups and experimental groups, which were treated with adipose­derived stem cells (ADSCs), amniotic membrane (AM), and a co-culture system. **(B)** The rat was positioned in a supine posture and securely held down on the table. **(C)** Left and right first molars of a rat prior to extraction. **(D)** Complete removal of the first molar. **(E)** On Day 21, two osseous flaws were intentionally made with a dental round bur after removing the tooth. On Day 50, animals were subjected to sacrificial procedures. **(F)** The premaxillary bone area was obtained for computed tomography. Reproduced with permission from “Periodontal rat animal model and osseous defects creation” by Pin-Hsien Wu et al., licensed under CC BY-NC-ND 4.0.

Hsien Wu et al. provide a brief overview of the experimental design Figure 4A. First, a ﬁrst molar extraction was performed in all animals (Figures 4B,D). Three weeks following extraction, the defect was filled with freshly generated bone (Figure 4C). Subsequently, a surgical procedure was performed to generate a two-wall intrabony defect measuring 2.6 × 2.0 × 2.0 mm (Figure 4E), and the materials were then inserted into the defect. Ultimately, the incision was sealed with cyanoacrylate gel. After creating a bone defect for a period of 21 days, the animals were euthanized, and their maxillae were collected (Figure 4F) (17).

#### Swamp rice rat

The swamp rice rat, scientifically known as *Oryzomys palustris*, is an indigenous American species that is predominantly found in the southern regions of the United States. Swamp rice rats have been utilized in research studies due to their susceptibility to periodontal disease and ability to mimic human inflammatory responses. These unique characteristics make them a valuable animal model for studying periodontitis. These animals are extremely prone to periodontal disease, which can start as early as 2 weeks old. At around 3 months of age, the gingival tissues experience swelling, the creation of pockets, the buildup of debris, and ulceration. Alveolar bone resorption underneath the gingiva leads to tooth separation and ultimately tooth loss (18). Mandibular teeth have a higher degree of plaque accumulation compared to maxillary teeth. Calculus and root-surface caries commonly manifest in elderly animals. Research has demonstrated that the occurrence of periodontal disease is influenced by dietary variables. In younger animals, the condition can be initiated by a soft-powder meal that has a large amount of carbohydrates. Conversely, diets that are heavy in fat or protein might lessen the severity of the infection (19). Initial pathological observations reveal an immediate inflammatory reaction, as polymorpho-nuclear cells infiltrate underneath the junctional and crevicular epithelium and into the gingival sulcus. This is then followed by the infiltration of “activated” macrophages into the afflicted epithelium. The destruction of connective tissues leads to the migration of epithelial attachment towards the apex of the root, resulting in the widening of the pocket (10). The advanced lesions manifest as the deterioration of the alveolar bone accompanied by fibrosis and the formation of granulation tissue in the gingival connective tissues and the periodontal ligament gap. Gram+ve bacteria. *S. sanguis, actinomyces*, and *Lactobacilli* have been identified in the oral cavity of rats aged 5 -9 weeks. In contrast to the gradual development of periodontitis in humans, the illness in rice rats rapidly leads to the long-term damage of both soft and hard tissues within a matter of weeks.

Rice rats have been utilized to assess the impact of food and certain treatment interventions. Additionally, the rapid progression of periodontitis in rice rats makes them a valuable model for studying the disease and potential treatments. By studying the effects of different interventions on rice rats, researchers gain valuable insights into how to prevent and treat periodontitis in humans (20).

#### Baker mouse model

As an outcome for the clinical presentation of periodontitis in humans, the Baker mouse model of periodontitis has been utilized to measure the alveolar bone resorption that is brought on by oral bacterial inoculums. This model has provided valuable insights into the pathogenesis of periodontitis and has been instrumental in testing potential therapeutic interventions. Overall, the Baker mouse model serves as a useful tool for studying the progression and treatment of periodontitis in a controlled laboratory setting (21). In order to evaluate the severity of periodontal pathogens, female BALB/c mice that were free of particular infections and were 10 weeks old, were infected orally with strains of *P. gingivalis*. Before being infected, mice were administered antibiotics Cotrimoxazole in their water for around 10 days to inhibit the growth of the usual microorganisms found in their mouths. The mice were subjected to oral gavage, receiving either a single kind of bacteria or a combination of bacteria mixed in carboxymethyl cellulose, in order to establish the infection. This treatment was administered five times, with a 2-day gap between each administration. Alveolar bone loss was seen after a period of 10 weeks.

#### Murine incisor abscess model

Rodent incisors have no roots and are continually erupting. In order to create a gum pocket abscess model, Institute of Cancer Research (ICR) mice (aged 3–6 weeks) were injected with *F. nucleatum*, a bacterium that does not usually inhabit mice, into the gums of their lower incisors for a period of 3 days. The injection of *F. nucleatum* led to the formation of abscesses around the lower incisors, mimicking the human condition of periodontal disease. This model allows for the study of infection and inflammation in a controlled laboratory setting. The swelling at the injection site suggested that *F. nucleatum* had caused a brief infection. The histological analysis, revealed the presence of granuloma development inside the inflamed gingiva. This model requires frequent administration of microorganisms and has a restricted application in researching gum pocket abscesses to simulate persistent halitosis induced by microbial infection (22). Overall, this model provides valuable insights into the progression and treatment of periodontal disease. However, its limitations in mimicking chronic conditions may necessitate further refinement for more comprehensive research on the long-term effects of gum infections.

#### Murine back abscess model

The murine back abscess model has been used to look into the connections between different types of oral pathogens and how the host reacts to them. This model has provided valuable insights into the pathogenesis of oral infections and potential treatment strategies. Additionally, it has been instrumental in studying the efficacy of various antimicrobial agents in treating these infections. This is especially useful for studying single-species infections that hurt soft tissue, like those caused by *P. gingivalis* and *Treponema denticola.* Researchers have found that when *P. gingivalis* and *F. nucleatum* or *P. gingivalis* and *A. actinomycetem comitans* get together, the abscesses get bigger than when they infect alone (23). The simultaneous presence of *S. constellatus* and *F. nucleatum* resulted in the death of mice, although the presence of either of these organisms alone did not induce mortality. Furthermore, the mouse subcutaneous chamber model has been used to study how the host and bacteria interact, as well as to find out how the different strains of *P. gingivalis* cause tissue damage and invasion. Despite the absence of lesions in the mouth cavity, this model is nevertheless useful for studying bacterially generated infections/co-infections that cause damage to soft tissues. This model allows researchers to better understand the pathogenic mechanisms of these bacteria and their interactions within the host (24).

#### 2.1.2 Rabbits

The analysis of oral microorganisms in rabbits revealed the presence of several harmful bacteria, such as *F. nucleatum, P. heparinolytica, Prevotella* spp., *P. micros, S. milleri* group, *A. israelii*, and *A. haemolyticum*. This finding is somewhat in line with the bacterial composition associated with periodontal disease in humans. Rabbits have been utilized in experiments involving surgically generated periodontal defects and the research of periodontal regeneration. However, they have been deemed less appropriate for the regeneration of the periodontal ligament. Rabbit models have provided valuable insights into the similarities and differences between rabbit and human oral microbiomes, shedding light on potential treatment options for periodontal disease in both species (25). Additionally, further research utilizing rabbits may help to better understand the mechanisms of periodontal regeneration and to improve treatment outcomes.

### 2.2 Clinically relevant animal models of periodontal disease

#### 2.2.1 Dogs

Periodontal disease is one of the most commonly reported dental inflammatory diseases in dogs. Like humans, periodontal disease in dogs can lead to gum infections, teeth loss, and serious health problems. Table 1 covers the key similarities and differences between dogs and humans regarding periodontal disease.

**Table 1:** Similarity in between various factors and presentation forms of periodontal disease in dogs and humans.

Aspect	Dogs	Humans
Etiology	*Porphyromonas gulae, Tannerella forsythia, Treponema denticola, Treponema putidum* (Kačírová J, 2022).	*Porphyromonas gingivalis, Treponema denticola*, and *Tannerella forsythia* are key pathogens (Mohanty R et al).
Prevalence	Common, affects around 80% of dogs over 3 years old.	Common, affects a large percentage of adults.
Transmission	Direct contact, shared objects, poor oral hygiene.	Person-to-person via saliva, poor oral hygiene.
Symptoms	Bad breath, inflamed gums, tooth loss, difficulty eating.	Bad breath, gum bleeding, receding gums, tooth loss.
Risk Factors	Age, small breed dogs, genetics, diet.	Age, smoking, genetics, diet.
Progression	Gingivitis to advanced periodontitis.	Gingivitis to advanced periodontitis.
Diagnosis	Veterinary dental exams, x-rays.	Dental exams, x-rays, probing.
Treatment	Professional cleaning, tooth extraction, antibiotics.	Professional cleaning, scaling, surgery, antibiotics.
Prevention	Regular brushing, dental treats, professional cleanings.	Regular brushing, flossing, professional cleanings.
Complications	Can lead to systemic diseases (heart, kidney issues).	Can lead to systemic diseases (heart disease, diabetes).

Dogs are naturally predisposed to periodontal disease, which affects the periodontium and can lead to complications such as gingivitis and varying degrees of periodontitis. As the condition advances, it becomes more severe, often resulting in tooth loss. Periodontal disease is highly prevalent in dogs, with reports by Harvey, C. (2022) indicating that approximately 80% of dogs and cats may be affected at some point in their lives. Albuquerque et al. (2012) reviewed canine periodontitis as a model for periodontal research. They described periodontal disease as an inflammatory condition caused by bacterial plaque that progresses from gingivitis to periodontitis through interactions between plaque microorganisms and host immune responses. The authors highlighted the high prevalence of periodontal disease in both humans and animals and emphasized the importance of early diagnosis and treatment. Among various animal models used to study periodontal disease, dogs were identified as one of the most valuable because their periodontal anatomy and disease progression closely resemble those in humans, making them useful for developing preventive and therapeutic strategies (26).

The use of dogs in periodontal research is often challenged by regulations regarding animal care, which require adequate companionship, exercise, space, and general upkeep. These regulations can hinder long-term studies on periodontal disease in dogs. Despite these obstacles, dogs remain a valuable model for exploring the progression and treatment of periodontal disease due to their genetic and physiological similarities to humans.

**Figure 5:**
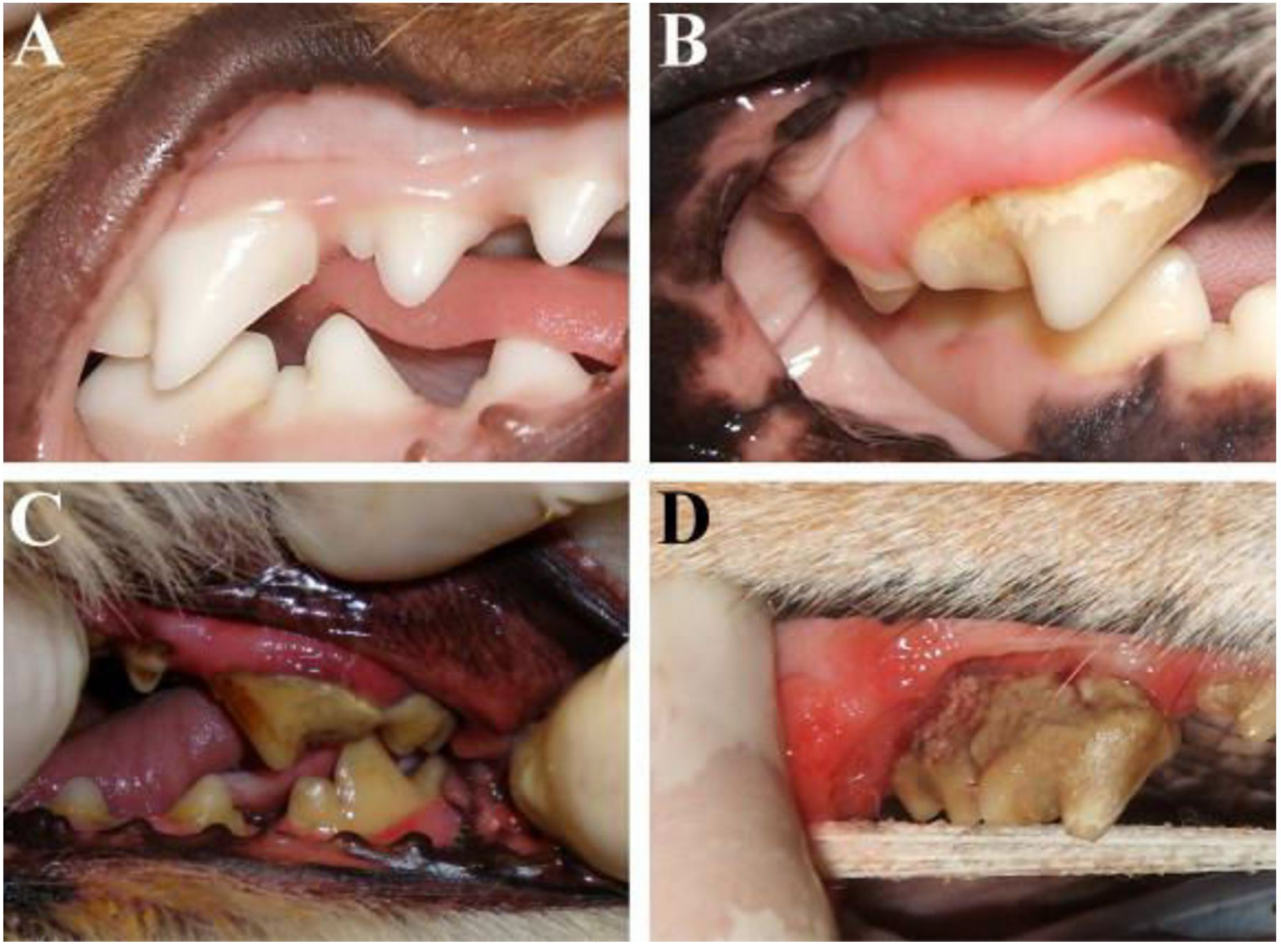
Illustrates the periodontium at various phases of both health and illness. **(A)** This shot depicts the upper and lower right arches of a dog's mouth, displaying healthy gum tissues. **(B)** Mild gingivitis; image of the upper right fourth premolar of a dog with a modest redness of the gum line and a small amount of hardened plaque on the teeth. **(C)** The dog's maxillary left fourth premolar has moderate gingivitis, as shown by notable redness and swelling of the gums, along with a growing buildup of calculus on the tooth. **(D)** Advanced periodontitis; image of the upper right fourth premolar in a dog with noticeable gum recession, intense gum inflammation, and a buildup of calculus. Reproduced with permission from “Periodontium at different stages of health/disease” by Carlos Albuquerque, Francisco Morinha, JoãoRequicha, Teresa Martins, Isabel Dias, HenriqueGuedes-Pinto, Estela Bastos, Carlos Viegas, licensed under CC BY-NC-ND 4.0.

Albuquerque et al. (2012) provided a detailed clinical description of gingiva health and disease progression in dogs. In a healthy dog, the gingiva is devoid of pigmentation, coral pink in colour, and exhibits a smooth, even texture. The gingival margin is sharp and well-defined, resembling the edge of a knife (Figure 5A). Early signs of inflammation, such as mild redness along the gum line, are indicative of slight gingivitis (Figure 5B). As the condition progresses, moderate inflammation are observed, marked by swelling and bleeding upon probing (Figure 5C). In advanced cases of gingivitis, the gums become severely inflamed, enlarged, or receded, with noticeable redness, swelling, and spontaneous bleeding (Figure 5D) (26).

The onset of periodontitis is characterized by the loss of attachment of periodontal tissues, accompanied by various pathological changes. These include the apical migration of the junctional epithelium, which leads to the formation of periodontal pockets, gingival recession, and alveolar bone resorption, contributing to the overall deterioration of the supporting structures of the teeth (27).

#### 2.2.2 Cats

According to studies, periodontal disease is among the most prevalent disorders in cats. Notably, different cat breeds exhibit varying levels of susceptibility to the disease. The condition is most commonly diagnosed in Siamese cats, with an incidence rate of 18.7%, followed by Maine Coons at 16.7%, British Shorthairs at 15.5%, and crossbreeds at 15.4%.

**Figure 6:**
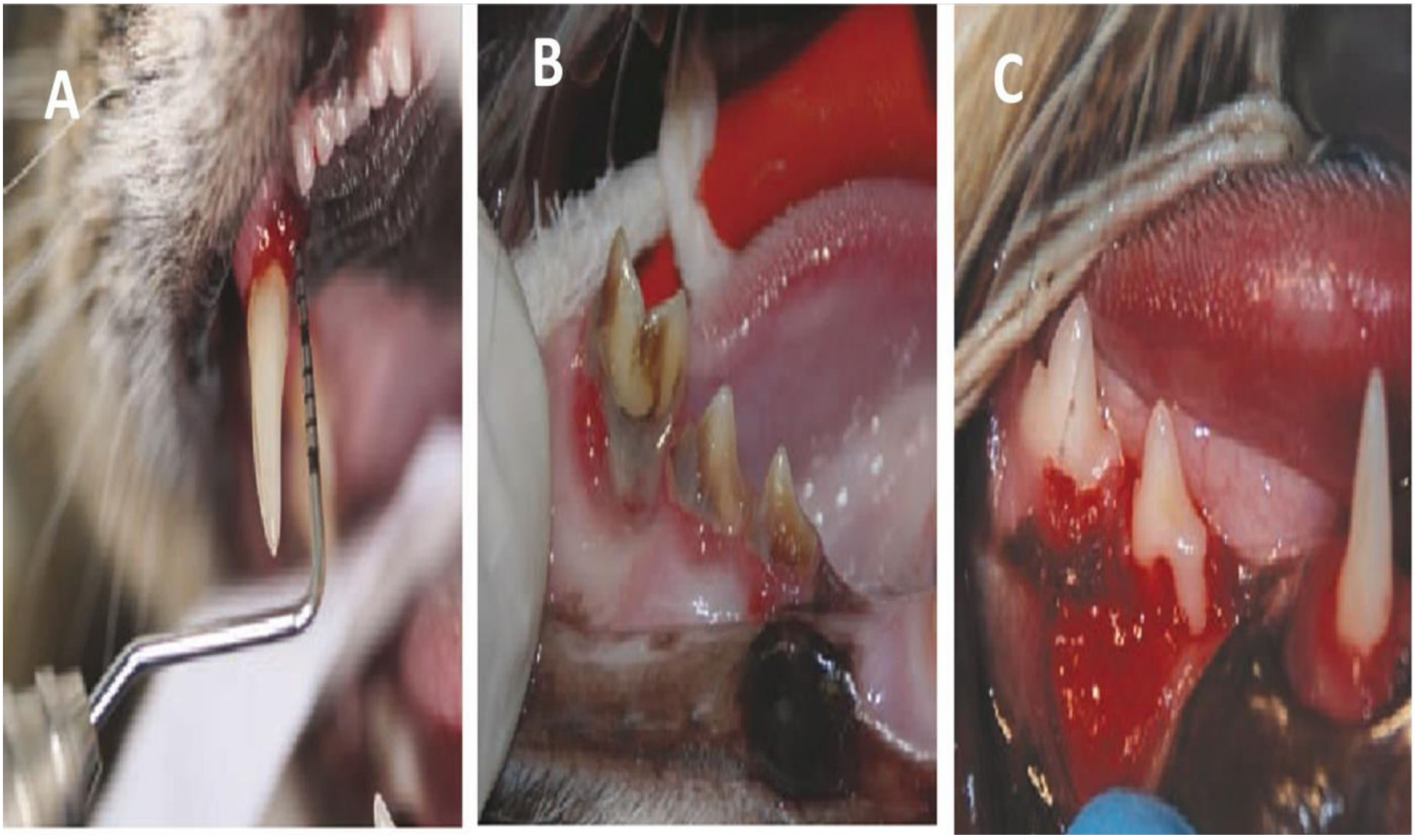
**(A)** Illustrates a significant periodontal pocket located on the palatal side of the upper right canine tooth. **(B)** The presence of dental calculus and inflammation of the gums are impacting the right lower third and fourth premolars and first molar. The right mandibular third premolar is affected by severe localized periodontitis. **(C)** There is a significant reduction of gum tissue and bone around the tooth, which has resulted in the exposure of a large portion of the outer surface of the root (known as resorption). Reproduced with permission from (A) “Deep periodontal pocket (6 mm) on the palatal aspect of the right maxillary canine (104)”, (B) “Severe periodontitis”, (C) “Severe focal periodontitis of the right mandibular third premolar (407)” by Rachel Perry and Cedric Tutt, licensed under CC BY 4.0.

Rachel Perry and Cedric identified a significant periodontal pocket located on the palatal aspect of the upper right canine teeth, indicative of severe periodontal disease (Figure 6A). The right lower third and fourth premolars, as well as the first molar, were affected by substantial dental calculus and gingival inflammation (Figure 6B). Additionally, severe localized periodontitis was observed in the right mandibular third premolar. This condition was characterized by a marked loss of gingival tissue and surrounding bone, resulting in the exposure of a large portion of the tooth's root surface, a phenomenon referred to as resorption (Figure 6C) (28).

#### 2.2.3 Nonhuman primates

Scientific studies indicate that humans diverged from primates, with molecular evidence suggesting that this separation occurred between 8 and 4 million years ago, when primates such as gorillas, and later chimpanzees, branched off to form distinct lineages, including that of humans. Human DNA is approximately 98.4% identical to that of chimpanzees (22). Histologically and anatomically, the structure of the periodontium in non-human primates (NHPs) is also closely aligned with that in humans. Certain NHP species develop periodontal diseases in adulthood, further emphasizing the biological parallels between the two groups.

Due to the anatomical and biological similarities between humans and NHPs, these primates are considered highly effective animal models for periodontal research. Their oral features and dentition closely resemble those of humans, enabling researchers to investigate the evolution of human dentition and oral health through studies on NHPs. Moreover, non-human primates offer valuable insights into the development and function of teeth in primates. NHPs also naturally develop dental plaque, calculus, oral microbial infections, such as *Porphyromonas gingivalis,* and periodontal disease, making them an important model for studying these conditions (29).

*Rhesus monkeys* (*Macaca mulatta*), *cynomolgus monkeys* (*Macaca fascicularis*), and *baboons* (*Papio anubis*) are inherently prone to periodontal disease. To speed up the periodontitis research, tools that make plaque, such as orthodontic elastic ligatures or sutures, are often placed below the interproximal area around some molars to help plaque formation. Ligatures are altered every 1–2 weeks until the presence of periodontal pocket development is established by probing, as shown by previous studies. Subsequently, the utilization of nonhuman primates was altered to encompass the administration of human diseases. *Cynomolgus monkeys* that did not show any prior signs of the human pathogen *P. gingivalis* were exposed to the organism. After about 5 months, the presence of *p. gingivalis* infection was verified and the development of plaque, which resulted in bone deterioration, was detected. While periodontitis in primates is the condition that most closely reflects the human illness, the high cost and specific care needs for these animals restrict their usage in periodontal research. Furthermore, they are susceptible to contagious illnesses such as TB, which diminishes their suitability as a model for periodontal disorders (30).

**Figure 7:**
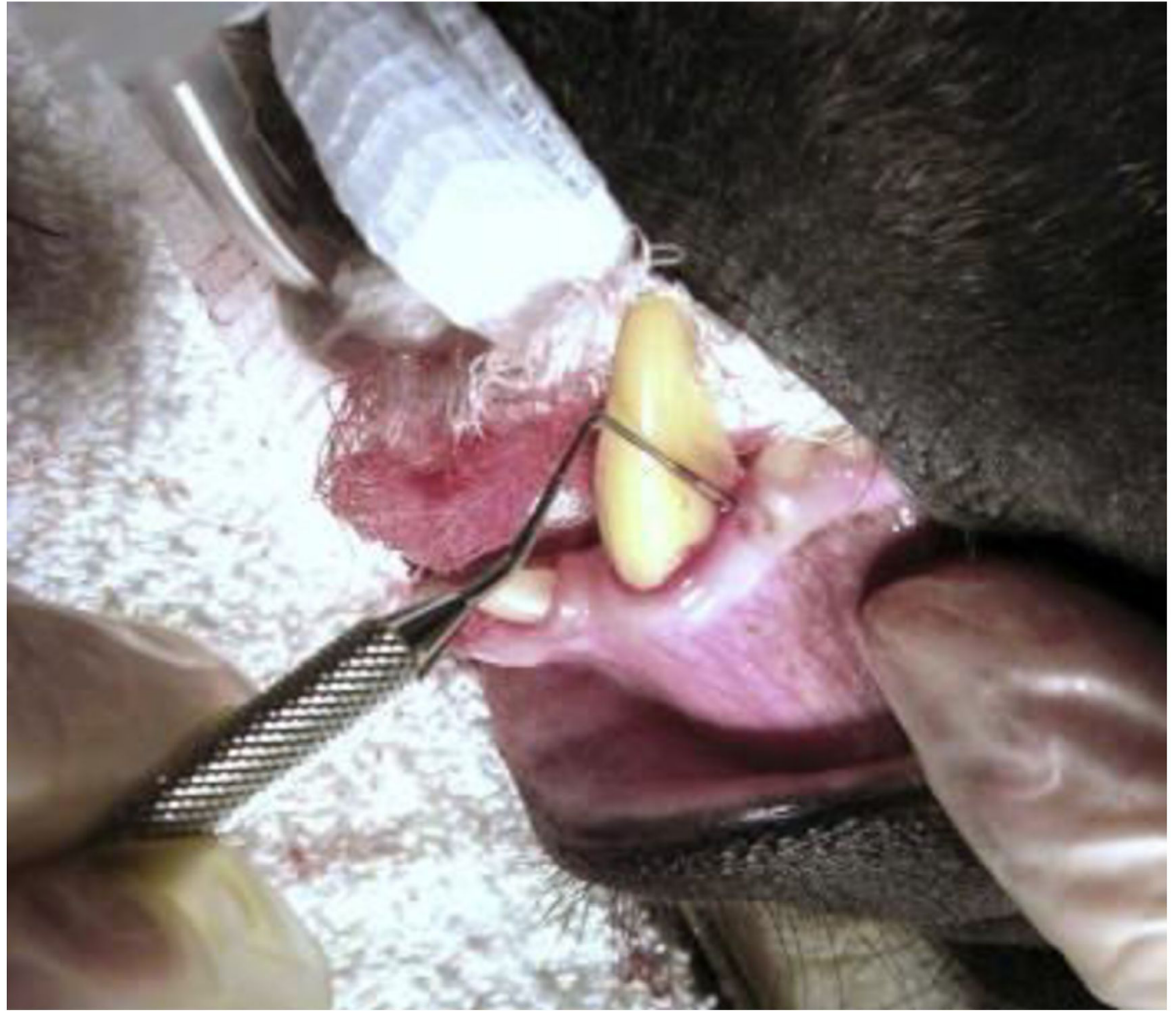
Illustrates examination of periodontal pockets and gums with dental probe in male Celebes monkey. Reproduced with permission from “Dental probe issued to check periodontal pockets and gums. This is an adult male Celebes macaque (Macacanigra)” by Cathy A. Johnson-Delaney, licensed under CC BY-NC-ND 4.0.

Delaney et al. describe how the sedated and intubated monkey may undergo simple blunting by using a low-speed dental drill with a finishing cone. To avoid thermally damaging the pulp, it is important to keep the tooth tip cold. Many male monkeys' canine teeth do not stay dull after coronal reduction; instead, regular canine occlusion sharpens the teeth, necessitating recurrent treatments. It should be mentioned that the male's canine teeth undergo self-sharpening and frequent wear, which causes them to become smaller in diameter with age. This natural process may require more frequent dental treatments to maintain the desired blunted shape (Figure 7). Additionally, regular monitoring and adjustments may be necessary to ensure the monkey's dental health and overall well-being (31).

In 2018, Jiang et al. conducted research utilising a monkey animal model to investigate periodontal examination. Diabetic monkeys and non-diabetic controls exhibited comparable levels of dental plaque and calculus at baseline. The control monkeys exhibited pink, resilient gingiva devoid of bleeding on probing. Furthermore, the probing depth was 2 mm and no attachment loss was observed, signifying that the periodontal tissues in the control group were healthy. At the start, diabetic monkeys had strong gums that didn't bleed when probed or lose their attachment (AL). However, a probing depth (PD) of 2–3 mm and mild swelling was observed.

Four months after the wire ligation, the diabetic rhesus monkeys' gingiva was slightly erythematous and oedematous, and they bled when probed to a depth of 3–4 mm (32). There was no significant attachment loss at the experimental sites. Nine months later, the rhesus monkeys in the diabetic cohort displayed erythematous and oedematous gingiva, pronounced bleeding on probing at the experimental sites, 4–7 mm deep periodontal pockets, and attachment loss at the majority of sites. Nine months post-wire ligation, the gingivae at the experimental sites of these diabetic monkeys exhibited erythema and oedema, with increased susceptibility to bleeding upon probing; additionally, periodontal pockets measuring 4–7 mm in depth, along with attachment loss at the majority of sites, were assessed.

Therefore researchers often turn to rodent models for studying periodontitis, as they are more cost-effective and easier to maintain in a controlled environment. Rodents can be genetically modified to mimic human conditions and are less susceptible to contagious diseases, making them a valuable alternative for studying periodontal disorders. Overall, rodent models have proven to be a reliable and efficient option for researchers looking to study periodontitis. Their ease of care and lower cost compared to primates make them a practical choice for many laboratories (33). Additionally, their genetic malleability allows for a more tailored approach to mimic human conditions, providing valuable insights into the mechanisms of periodontal diseases. With these advantages, rodent models continue to be a popular choice in the field of periodontal research.

#### 2.2.4 Miniature pigs

The Minnesota small pig, sometimes known as the minipig, was employed around six decades ago and has since been widely used in the field of scientific research. Minipigs often acquire gingivitis after reaching 6 months of age. This is characterized by inflammation of the gum tissue, the presence of plaque and calculus, and bleeding upon probing. Miniature pigs have been used as a substantial animal model in medical research due to scientific, economic, and ethical rationales. The oral maxillofacial area of tiny pigs has anatomical, developmental, physiological, pathophysiological, and disease occurrence similarities to those of humans. These similarities make miniature pigs valuable for studying various oral health conditions and developing new treatments. Researchers can utilize this animal model to gain insights that may ultimately benefit human patients (34).

Minipigs often get gingivitis at six months of age, which is characterized by inflammatory gingival tissue, a buildup of plaque and calculus, and bleeding upon probing. The gingival tissue is infiltrated by inflammatory cells, which at 16 months of age progresses to severe periodontal inflammation with a histology that is exactly like that of humans. Minipigs can get periodontitis in about 4–8 weeks after being fitted with ligatures and given bacteria such *as P. gingivalis, S. mutans, and A. actinomycetemcomitans.* Minipigs can be used for both orofacial and periodontal research (34).

**Figure 8:**
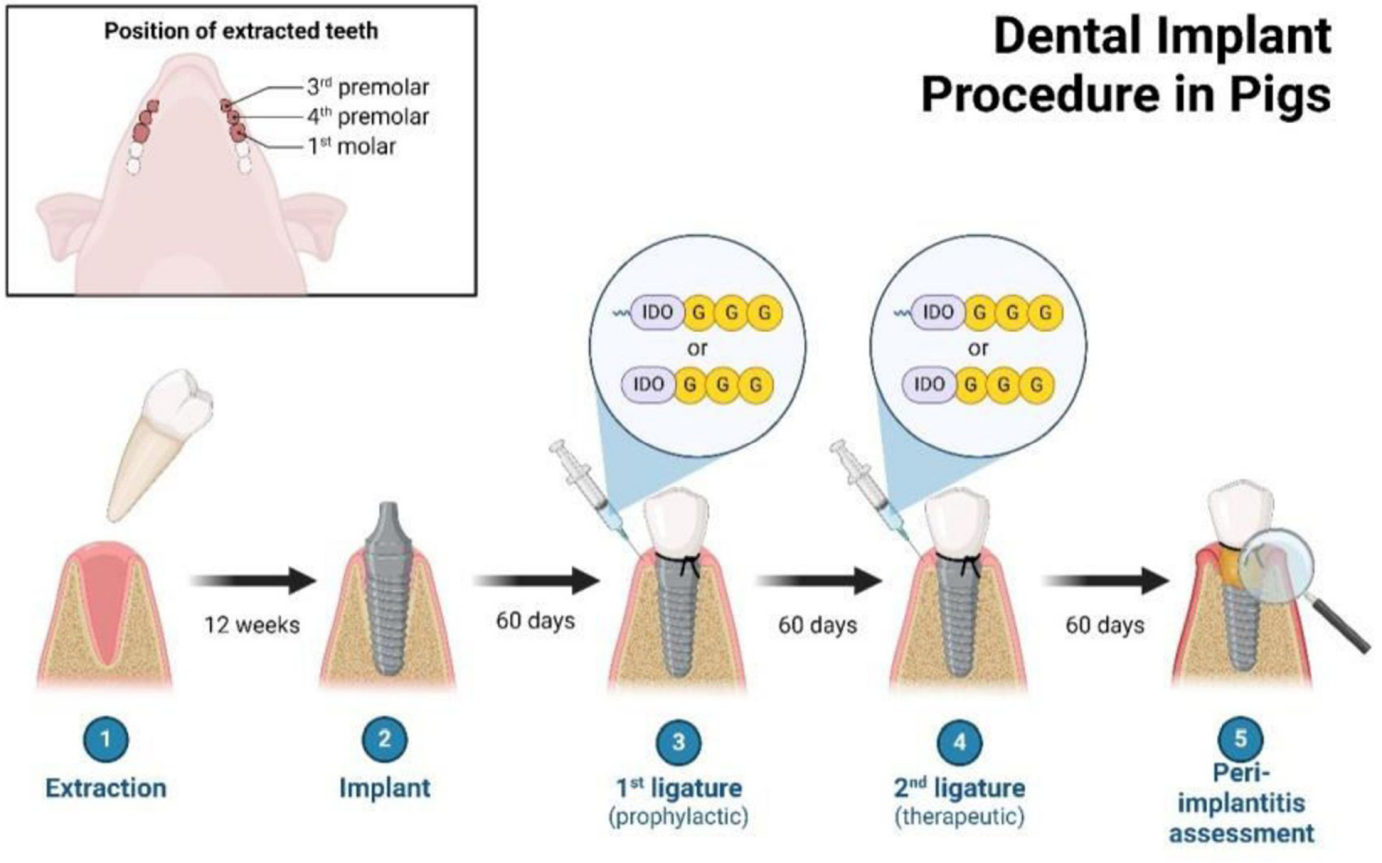
Illustrates the dental implant technique administered to a pig. Reproduced with permission from “Dental Implant Procedure in Pigs” by Christina Graves. Retrieved from https://app.biorender.com/biorender-templates.

The dental implant procedure is shown on a pig model to demonstrate the process before it is performed on humans. The pig model allows for practice and refinement of technique to ensure successful outcomes in human patients. This preclinical testing helps to minimize risks and complications during the actual procedure on human patients. Additionally, it provides valuable insights for improving the overall success rate of dental implant surgeries. The implant procedure is shown on a pig in Figure 8.

Additionally, their size and ease of handling make them a practical choice for researchers looking to study oral and periodontal health. With continued investment and research, minipigs have the potential to play a significant role in advancing our understanding of periodontal disease and developing new treatment strategies.

## 3 Other animal models

### 3.1 Horses

Prevalent oral illnesses that naturally occur in horses include buccal abrasions, calculus, gingival recession, and periodontal pockets. According to a recent assessment on horses, the occurrence of periodontal pockets and gingival recession is more common in older horses and is mostly linked to other dental problems and tooth loss. Due to their large size and the complexities involved in caring for them, horses are not a suitable choice for conducting fundamental scientific investigations on periodontitis or for evaluating prospective treatments. However, studying oral health in horses can still provide valuable insights into the development and progression of periodontal diseases in other species, including humans (35). By understanding how these conditions manifest in horses, researchers can potentially improve treatment options for all animals suffering from similar oral ailments.

### 3.2 Ferrets

Ferrets (Mustela putorius furo) naturally experience the development of calculus and periodontal disease, which is comparable to that in humans. In contrast to rodents, ferrets' ability to form calculus is unaffected by their diet and is testable in live animals. Ferrets serve as a suitable model for studying calculus; however, they have a tendency to easily escape from standard cages and require special care. Ferrets are a valuable model for studying periodontal disease and calculus formation due to their similarities to humans in this regard (36). However, researchers should be aware of the challenges associated with their care and containment in laboratory settings.

### 3.3 Hamsters

Hamsters possess a dental formula that closely resembles that of rats, and they may induce experimental periodontitis by applying ligatures to their molar teeth. Furthermore, hamsters possess buccal pouches that are lined with stratified squamous epithelium, making them valuable for researching oral cancer (37). The progression of the infection closely resembles that of rats. Additionally, hamsters are known for their ability to develop tumours in their cheek pouches, providing researchers with a valuable model for studying oral cancer progression (38). Overall, the similarities between hamsters and humans in dental structure and disease progression make them useful animal model for various research studies.

Various animal models are used in periodontal disease research, each with distinct advantages and limitations. Rodents (rats and mice) are frequently used due to their cost-effectiveness, short life cycles, and ease of handling, although they exhibit limited anatomical similarity to humans and rapid healing rates, complicating long-term studies. Non-human primates, while offering the closest anatomical and immunological resemblance to humans, are costly, ethically challenging and require long study durations. Dogs, which share similar tooth structures with humans, are widely used for dental research but are expensive and present ethical concerns due to their longer lifespan. Hamsters, on the other hand, offer quick disease induction at low cost, but their small size and limited resemblance to human periodontal structures pose challenges for surgical studies. Miniature pigs have a similar jaw and tooth structure to humans, making them suitable for long-term studies, but high costs, ethical concerns, and complex housing requirements limit their use (39). Rabbits, though easy to handle and capable of mimicking alveolar bone loss, are less commonly used due to their differing dental anatomy. Ferrets, with alveolar bone structures similar to humans, are useful for studying bone loss but are less common in research and require more maintenance. Sheep and goats offer advantages for bone regeneration studies due to their similar jawbone structure to humans, but their large size, high cost, and ethical concerns make them less feasible. Finally, guinea pigs, widely used for immune response studies, are cost-effective and exhibit rapid disease progression, but their poor dental anatomy limits their suitability for mimicking human periodontal conditions.

The Table 2 provides a comparison of various animal models used to induce periodontal disease, detailing the infection process, time taken to develop the condition, feasibility of the model, and the associated advantages and disadvantages of each animal model. It serves as a guide to select the appropriate model based on study requirements, cost, ethical concerns, and anatomical similarity to humans.

**Table 2:** Comparison of animal models for inducing periodontal disease.

Animal Model	Process to produce infection	Time taken	Feasibility	Advantages	Disadvantages
Rodents (Rats, Mice)	Inoculation with periodontal pathogens (e.g., *P. gingivalis*, *A. actinomycetem-comitans*)	4–8 weeks	High	Cost-effective, short life cycle, easy to handle, well-established methods.	Limited anatomical similarity to human periodontium, rapid healing rates, ethical concerns over long-term studies.
Non-Human Primates	Placement of ligatures around teeth, inoculation with pathogens	3–6 months	Moderate to Low	Closest anatomical and immunological similarity to humans, mimics human periodontal disease progression.	Expensive, ethical concerns, long study durations, complex husbandry.
Dogs	Placement of ligatures, naturally occurring periodontal disease	3–12 months	Moderate	Similar tooth structure to humans, natural progression of periodontal disease, widely used in dental research.	Ethical concerns, expensive, longer lifespan compared to rodents, harder to maintain in large studies.
Hamsters	Dietary modification (high carbohydrate diet) and pathogen inoculation	2–4 weeks	High	Quick disease induction, low cost, easy handling.	Limited resemblance to human periodontium, small size making surgical procedures difficult.
Miniature Pigs	Ligature-induced periodontitis, pathogen inoculation	6–12 months	Low	Similar jaw and tooth structure to humans, long-term disease progression similar to humans.	High cost, ethical concerns, difficult housing and handling.
Rabbits	Inoculation with *A. actinomycetem-comitans* or *P. gingivalis*	6–10 weeks	Moderate	Can mimic alveolar bone loss, relatively easy to handle.	Not as widely used, dental anatomy not closely aligned with humans.
Ferrets	Ligature placement, pathogen inoculation	4–8 weeks	Moderate	Alveolar bone structure similar to humans, good for studying alveolar bone loss.	Less common in research, higher maintenance requirements, ethical concerns.
Sheep/Goats	Ligature-induced periodontitis, inoculation	3–9 months	Low	Similar jawbone structure, good for bone regeneration studies.	Large size makes them difficult to house, high cost, ethical concerns.
Guinea Pigs	Pathogen inoculation, ligature placement	3–6 weeks	High	Cost-effective, rapid progression of periodontitis, widely used for immune response studies.	Poor dental anatomy for mimicking human periodontium, smaller size limits surgical studies.

Further by a thorough comparison of results from animal studies along with clinical observations, researchers can better understand the similarities and differences between species in terms of periodontal disease progression. This comparative approach helps to bridge the gap between animal models and human applications in periodontal research (40). Ultimately, the goal is to develop a reliable animal model that closely mimics the pathogenesis of periodontal disease in humans. This would allow researchers to devise potential preclinical treatments and interventions (41). By establishing a suitable animal model, researchers can potentially accelerate the development of new therapies for periodontal disease and improve outcomes for patients.

## 4 Conclusion

Every animal model used to study periodontal disease has its own set of advantages and limitations. Many models demonstrate resemblances to human illnesses. Although non-human primates have the greatest similarities with humans, their high expense and challenges in caring for them make it impractical to utilize them extensively in both fundamental scientific research and treatment investigations on periodontal disease. While rodents are more cost-effective and simpler to manage, they do not fully replicate the course of periodontitis in humans. For instance, it is sometimes necessary to use ligatures and/or introduce external (human) pathogens, which are just a small fraction of the hundreds of bacteria that make up dental plaque biofilm, in order to cause illness. Furthermore, rodents possess distinct dental anatomical variations. However, rats and mice are valuable for comprehending some facets of the relationship between hosts and microbes, as well as for studying therapeutics. Overall, while rodents may not perfectly mimic human periodontal disease, they still provide valuable insights into the disease process and potential treatments. Their use in research allows for a better understanding of the complex interactions between bacteria and host immune responses in the development of periodontitis. The continued exploration of different animal models is crucial in advancing our understanding of periodontal disease and finding new ways to prevent and treat this common oral health condition.
