# Correction: Insights into periodontal disease: comparative analysis of animal models

**DOI:** 10.3389/fdmed.2026.1823139

**Published:** 2026-06-25

**Authors:** Binapani Barik, Saurabh Chawla, Bhabani Sankar Satapathy, Swadesh Kumar Pattanik, J. Aravind Kumar, Saleh Al-Farraj, Gurudutta Pattnaik, Mika Sillanpää

**Affiliations:** 1 School of Pharmacy and Life Sciences, Centurion University of Technology and Management, Khordha, Odisha, India; 2 School of Biological Sciences, National Institute of Science Education and Research (NISER), Khordha, Odisha, India; 3 Homi Bhabha National Institute (HBNI), Training School Complex, Anushaktinagar, Mumbai, India; 4 GITAM School of Pharmacy, GITAM Deemed to be University, Hyderabad Campus, Telangana, India; 5 Chitkara College of Pharmacy, Chitkara University, Punjab, India; 6 Saveetha School of Engineering, Saveetha Institute of Medical and Technical Sciences, Saveetha University, Chennai, Tamil Nadu, India; 7 College of Science, King Saud University, Riyadh, Saudi Arabia; 8 Saveetha School of Engineering, Saveetha Institute of Medical and Technical Sciences, Saveetha University, Chennai, Tamil Nadu, India; 9 Centre of Research Impact and Outcome, Chitkara University Institute of Engineering and Technology, Chitkara University, Rajpura, Punjab, India

**Keywords:** periodontal disease, animal model, bacterial infection, comparative study, limitations

Affiliation 5 was erroneously given as “5. Amity School of Pharmacy, Amity University, Noida, Uttar Pradesh, India”. It should be “5. Chitkara College of Pharmacy, Chitkara University, Punjab, India”.

In the published article, several references were listed incorrectly.

The reference 4 was erroneously written as “Fernández-Bravo, S. E. (2022). Antioxidants in Dentistry: Oxidative Stress and Periodontal Diseases. In Lipid Oxidation in Food and Biological Systems: A Physical Chemistry Perspective (pp. 341–359). Cham: Springer International Publishing.” It should be “Saluja, H. M., Sachdeva, S., & Mani, A. (2021). Role of reactive oxygen species and antioxidants in periodontal disease. Journal of Cellular Biotechnology, 7(2), 125–140”.

The reference 12 was erroneously written as “Harvey C. The relationship between periodontal infection and systemic and distant organ disease in dogs. Vet Clin Small Anim Prac. (2022) 52(1):121–37. doi: 10.1016/j.cvsm.2021.09.004”. It should be “Yin, D., Zhan, S., Liu, Y. et al. Experimental models for peri-implant diseases: a narrative review. Clin Oral Invest 28, 378 (2024). https://doi.org/10.1007/s00784-024-05755-7”.

The reference 13 was erroneously written as “da Cunha, E. S. G. (2021). Periodontal Disease in Dogs: An Experimental Approach Towards Prevention Using Antimicrobial Peptides (Doctoral dissertation, Universidade de Lisboa (Portugal))”. It should be “Tomina, D. C., Petruțiu, Ș. A., Dinu, C. M., Crișan, B., Cighi, V. S., & Rațiu, I. A. (2022). Comparative testing of two ligature-induced periodontitis models in rats: a clinical, histological and biochemical study. Biology, 11(5), 634”.

The reference 14 was erroneously written as “Deng, J., Golub, L. M., Lee, H. M., Lin, M. C., Bhatt, H. D., Hong, H. L., ..& Gu, Y. (2020). Chemically-modified curcumin 2.24: a novel systemic therapy for natural periodontitis in dogs. Journal of Experimental Pharmacology, 47-60”. It should be “Basic, A. and Dahlén, G., 2023. Microbial metabolites in the pathogenesis of periodontal diseases: a narrative review. Frontiers in Oral Health, 4, p.1210200”.

The reference 15 was erroneously written as “Jepsen, K., Sculean, A., & Jepsen, S. (2023). Complications and treatment errors related to regenerative periodontal surgery. Periodontology 2000, 92(1), 120–134”. It should be “Rose, R.K., 2023. Oryzomys palustris (Rodentia: Cricetidae). Mammalian Species, 55(1031), p.sead006 Applied Pharmaceutics. 2019;11(4):172–4”.

The reference 16 was erroneously written as “Wu, P. H., Chung, H. Y., Wang, J. H., Shih, J. C., Kuo, M. Y. P., Chang, P. C., .. & Chang, C. C. (2016). Amniotic membrane and adipose-derived stem cell coculture system enhances bone regeneration in a rat periodontal defect model. Journal of the Formosan Medical Association, 115(3), 186–194”. It should be “Dharmawati IG. Pocket measurement methods in wistar rats periodontitis induced by bacteria and the installation of silk ligature: An experimental studies. International Journal of Applied Pharmaceutics. 2019;11(4):172–4”.

The reference 18 was erroneously written as “Alanbari, B.F. and Al-Taweel, F.B.H., 2024. Animal models in periodontal research: A narrative review. Journal of Emergency Medicine, Trauma & Acute Care, 2024(2), p.5”. It should be “Gui, Q., Lyons, D. J., Deeb, J. G., Belvin, B. R., Hoffman, P. S., & Lewis, J. P. (2021). Non-human primate Macacamulatta as an animal model for testing efficacy of amixicile as a targeted anti-periodontitis therapy. Frontiers in Oral Health, 2, 752929”.

The reference 19 erroneously written as “Abdul-Azees, P. A., Wang, H., Chun, Y. H. P., Pizzini, J., Dean, D. D., Reveles, K. R., .. &Yeh, C. K. (2024). Changes in oral health during aging in a novel nonhuman primate model. GeroScience, 46(2), 1909–1926”. It should be “Frazão, D.R., Santos Mendes, P.F., Baia-da-Silva, D.C., Mendonça de Moura, J.D., Neves dos Santos, V.R., Matos-Sousa, J.M., de Souza Balbinot, G., Guimarães, D.M., Collares, F.M. and Lima, R.R., 2023. Modulation of blood redox status by the progression of induced apical periodontitis in rats. Frontiers in physiology, 14, p.1214990”.

The reference 20 was erroneously written as “Son SJ, Jang S, Rah H, Choi S. Characteristics of the dental pulp and periodontal ligament stem cells of the Yucatan Miniature Pig. Appl Sci. (2021) 11(20):9461. doi: 10.3390/app11209461”. It should be “Guvva S, Patil MB, Mehta DS. Rat as laboratory animal model in periodontology. International Journal of Oral Health Sciences. 2017 Jul 1;7(2):68-75”.

The reference 21 was erroneously written as “Li G, Han N, Yang H, Zhang X, Cao Y, Cao Y, et al. SFRP2 Promotes stem cells from apical papilla-mediated periodontal tissue regeneration in miniature pig. J Oral Rehabil. (2020) 47:12–8. doi: 10.1111/joor.12882”. It should be “Oz, H.S. and Puleo, D.A., 2011. Animal models for periodontal disease. BioMed Research International, 2011(1), p.754857”.

The reference 25 was erroneously written as “Nagao Y, Takahashi H, Kawaguchi A, Kitagaki H. Effect of fermented rice drink “Amazake” on patients with nonalcoholic fatty liver disease and periodontal disease: a pilot study. Reports. (2021) 4(4):36. doi: 10.3390/reports4040036”. It should be “Flenghi L, Mazouffre M, Le Loc'h A, Le Loc'h G, Bulliot C. Normal bacterial flora of the oral cavity in healthy pet rabbits (Oryctolagus cuniculus). Veterinary Medicine and Science. 2023 Jul;9(4):1621–6”.

The reference 27 was erroneously written as “Arrazuria, R., Knight, C. G., Lahiri, P., Cobo, E. R., Barkema, H. W., & De Buck, J. (2020). Treponema spp. isolated from bovine digital dermatitis display different pathogenicity in a murine abscess model. Microorganisms, 8(10), 1507”. It should be “Stepaniuk, K., 2019. Periodontology. Wiggs's Veterinary Dentistry: Principles and Practice, pp.81–108”.

The reference 28 was erroneously written as “Bhattarai G, Poudel SB, Kook SH, Lee JC. Resveratrol prevents alveolar bone loss in an experimental rat model of periodontitis. Actabiomaterialia. (2016) 29:398–408.”. It should be “Perry R, Tutt C. Periodontal disease in cats: back to basics-with an eye on the future. J Feline Med Surg. (2015) 17(1):45–65. doi: 10.1177/1098612X14560099”.

The reference 33 erroneously written as “Zakaria, M. F., Sonoda, S., Kato, H., Ma, L., Uehara, N., Kyumoto-Nakamura, Y., ..&Yamaza, T. (2024). Erythropoietin receptor signal is crucial for periodontal ligament stem cell-based tissue reconstruction in periodontal disease. Scientific reports, 14(1), 6719”. It should be written as “Hajishengallis, G., Lamont, R.J. and Graves, D.T., 2015. The enduring importance of animal modelsin understanding periodontal disease. Virulence, 6(3), pp.229–235”.

The reference 34 erroneously written as “Hernández Martínez, C. D. J., Felix Silva, P., Salvador, S. L., Messora, M., &Palioto, D. B. (2023). Chronological analysis of periodontal bone loss in experimental periodontitis in mice. Clinical and Experimental Dental Research, 9(6), 1009–1020”. It should be “Lunney, J.K., Van Goor, A., Walker, K.E., Hailstock, T., Franklin, J. and Dai, C., 2021. Importance of the pig as a human biomedical model. Science translational medicine, 13(621), p.eabd5758”.

The reference 36 erroneously written as “Scott, C. (2023). Investigating the virulence of Treponema phagedenis strains isolated from digital dermatitis lesions in a murine abscess model”. It should be written as “Johnson-Delaney, C.A., 2016. Anatomy and disorders of the oral cavity of ferrets and other exotic companion carnivores. Veterinary Clinics: Exotic Animal Practice, 19(3), pp.901–928.”

The reference 37 erroneously written as “Varela-López, A., Bullón, P., Ramírez-Tortosa, C. L., NavarroHortal, M. D., Robles-Almazán, M., Bullón, B., .&Quiles, J. L. (2020). A diet rich in saturated fat and cholesterol aggravates the effect of bacterial lipopolysaccharide on alveolar bone loss in a rabbit model of periodontal disease. Nutrients, 12(5), 1405.” It should be written as “El-Ghareeb, M.H., 2004. Human oral mucosal transplants into the hamster cheek pouch (Master's thesis, The Ohio State University”.

The reference 38 was erroneously written as “Zapf, A. M., Fey, K., Büttner, K., Gröf, M., &Staszyk, C. (2023). Periodontal structures in horses with pituitary pars intermedia dysfunction: A histological evaluation. Frontiers in Veterinary Science, 10, 1114445”. It should be “Khayatan, D., Hussain, A. and Tebyaniyan, H., 2023. Exploring animal models in oral cancer research and clinical intervention: A critical review. Veterinary Medicine and Science, 9(4), pp.1833–1847”.

The reference 39 was erroneously written as “Mustafa, H., Cheng, C. H., Radzi, R., Fong, L. S., Mustapha, N. M., &Dyary, H. O. (2021). Induction of periodontal disease via retentive ligature, lipopolysaccharide injection, and their combination in a rat model. Polish journal of veterinary sciences, 365–373.” It should be “Nokhbatolfoghahaei, H., Paknejad, Z., Bohlouli, M., Rezai Rad, M. and Khojasteh, A., 2019. Animal models in dental research. In Applications of biomedical engineering in dentistry (pp. 377–442). Cham: Springer International Publishing.”

The reference “Oz, H.S. and Puleo, D.A., 2011. Animal models for periodontal disease. BioMed Research International, 2011(1), p.754857” was not cited in the article. The citation has now been inserted in the “*Baker mouse model*” section as reference 21. The text should now read: “Overall, the Baker mouse model serves as a useful tool for studying the progression and treatment of periodontitis in a controlled laboratory setting (21).”

An incorrect number was provided in the Funding statement for Project number “(RSP2025R140)”. The correct Project Number is “(RSP2025R7)”. The full Funding statement should now read:

“The author(s) declare that financial support was received for the research and/or publication of this article. APC covered by Researchers Supporting Project Number (RSP2025R7) King Saud University, Riyadh, Saudi Arabia. Researchers acknowledge Supporting Project number (RSP2025R7), King Saud University, Riyadh, Saudi Arabia for the support to execute the work.”

A correction has been made to the section **3 Other animal models**, *3.2 Ferrets*. “Ferrets (*Mustela putorius*) naturally experience the development of calculus and periodontal disease, which is comparable to that in humans.” should now read “Ferrets (*Mustela putorius furo*) naturally experience the development of calculus and periodontal disease, which is comparable to that in humans.”

In section **2 Models based on animals**, *2.2 Clinically relevant animal models of periodontal disease, 2.2.3 Nonhuman primates*, “Rhesus monkeys (Macaca mulatta), cynomolgus monkeys (Macaca fascicularis), and baboons (Papio anubis) are inherently prone to periodontal disease” is now italicized to “*Rhesus monkeys (Macaca mulatta), cynomolgus monkeys (Macaca fascicularis)*, and *baboons (Papio anubis)* are inherently prone to periodontal disease”.

A correction has been made to the reference citations for reference 26 and 27 in section 2.

A correction has been made to the section **2 Models based on animals**, *2.2 Clinically relevant animal models of periodontal disease , 2.2.1 Dogs,* paragraph 2. “These animals also serve as valuable models for studying the relationship between oral and systemic health in humans, as their oral microbiome closely resembles that of humans (26).” is now “Albuquerque et al. (2012) reviewed canine periodontitis as a model for periodontal research. They described periodontal disease as an inflammatory condition caused by bacterial plaque that progresses from gingivitis to periodontitis through interactions between plaque microorganisms and host immune responses. The authors highlighted the high prevalence of periodontal disease in both humans and animals and emphasized the importance of early diagnosis and treatment. Among various animal models used to study periodontal disease, dogs were identified as one of the most valuable because their periodontal anatomy and disease progression closely resemble those in humans, making them useful for developing preventive and therapeutic strategies (26)”.

In section **2 Models based on animals**, *2.2 Clinically relevant animal models of periodontal disease , 2.2.1 Dogs, *paragraph 4, the sentence previously read: “In advanced cases of gingivitis, the gums become severely inflamed, enlarged, or receded, with noticeable redness, swelling, and spontaneous bleeding (Figure 5D) (27).”

This now reads: “In advanced cases of gingivitis, the gums become severely inflamed, enlarged, or receded, with noticeable redness, swelling, and spontaneous bleeding (Figure 5D) (26).”

In section **2 Models based on animals**, *2.2 Clinically relevant animal models of periodontal disease , 2.2.1 Dogs, *paragraph 5, reference 27 is now cited:

“The onset of periodontitis is characterized by the loss of attachment of periodontal tissues, accompanied by various pathological changes. These include the apical migration of the junctional epithelium, which leads to the formation of periodontal pockets, gingival recession, and alveolar bone resorption, contributing to the overall deterioration of the supporting structures of the teeth (27).”

In section **2 Models based on animals***, 2.1 Experimentally induced animal models of periodontal disease, 2.1.1 Rodent models*, *Rats*. This previously read: “Rats are frequently employed in experimental models of periodontitis due to the similarities between the periodontal architecture in the molar area of rats and humans. The rice rat (Oryzomys palustris) is an omnivorous rodent with a diet primarily composed of plant materials and small animals. Its food sources include seeds, grains—particularly rice in cultivated regions—fruits, and green vegetation.”

The corrected sentences appear below:

“Rats are frequently used in experimental models of periodontitis due to similarities in periodontal architecture with humans, particularly in the molar region. Among rat models, the rice rat (*Oryzomys palustris*) is especially valuable due to its high susceptibility to naturally occurring and diet-induced periodontal disease, making it a suitable model for studying disease progression.”

The original version of this article has been updated.

